# Assessing the Usability, Appeal, and Impact of a Web-Based Training for Adults Responding to Concerning Posts on Social Media: Pilot Suicide Prevention Study

**DOI:** 10.2196/14949

**Published:** 2020-01-20

**Authors:** Bradley Kerr, David Stephens, Daniel Pham, Thomas Ghost Dog, Celena McCray, Colbie Caughlan, Amanda Gaston, Jesse Gritton, Marina Jenkins, Stephanie Craig Rushing, Megan A Moreno

**Affiliations:** 1 Department of Pediatrics University of Wisconsin-Madison Madison, WI United States; 2 Northwest Portland Area Indian Health Board Portland, OR United States; 3 Paul G Allen School of Computer Science and Engineering University of Washington Seattle, WA United States; 4 WithinReach Seattle, WA United States

**Keywords:** community health education, mental health, social networking

## Abstract

**Background:**

Suicide prevention remains challenging among youth, as many do not disclose suicidal ideation. Nearly one-third of American Indian and Alaska Native (AI and AN, tribal, or native) youth see concerning messages on social media at least weekly.

**Objective:**

To prepare adults to support AI and AN youth who post or view concerning messages, our team designed an hour-long training: Responding to Concerning Posts on Social Media. This study tested the usability, appeal, and impact of the training.

**Methods:**

A purposive sample of 70 adults was recruited to participate in the pilot, which included 2 study arms. Arm 1 participants completed a 30-min training video and reviewed accompanying handouts, including the Viewer Care Plan (VCP). The VCP provided a 3-step planning and response tool: (1) Start the Conversation, (2) Listen, Gather Information, and Assess Viewer Experience, and (3) Plan and Act. The intent of the VCP was to support and connect AI and AN youth who either view or post concerning messages on social media to life-saving resources. Those enrolled in arm 2 participated in an additional interactive role-play scenario with a coach that took place after the training, via text message. Participants provided qualitative and quantitative feedback on the training’s relevance, appeal, and utility. Paired *t* tests were used to assess confidence in addressing concerning posts between pre- and postsurveys. Content analysis of the role-play transcripts was used to assess the quality and completion of the coached role-plays, in relation to the recommended VCP.

**Results:**

Altogether, 35 participants finished the training and completed pre- and postsurveys; 22 participants completed the 6-month follow-up survey. Pre-post analyses of differences in means found significant improvement across several efficacy measures, including confidence starting a conversation about social media (*P*=.003), confidence contacting the person who posted something concerning (*P*<.001), and confidence recommending support services to youth who view (*P*=.001) or youth who post concerning messages (*P*<.001). Similarly, pre- to 6-month analyses found significant positive improvement across multiple measures, including confidence contacting the youth who posted (*P*<.001), confidence starting a conversation about social media with youth (*P*=.003), and an increase in the number of experiences recommending resources for youth who viewed concerning social media posts (*P*=.02). Of the 3 steps of the VCP, the least followed step in coached role-plays was sharing tools and resources, which is a part of the third Plan and Act step.

**Conclusions:**

Findings indicate that the Responding to Concerning Posts on Social Media training is a promising tool to prepare adults to intervene and complete the VCP. Additional evaluation with a larger cohort of participants is needed to determine the unique impact of the role-play scenario and changes in mental health referral rates, behaviors, and skills.

## Introduction

### Background

According to the Center for Native American Youth, suicide prevention remains one of the most daunting challenges for American Indian and Alaska Native (AI and AN, tribal, or native) communities [1]. Among Native youth in 9th to 12th grades, including teens aged 14-18 years, the past year prevalence of suicidal thoughts, suicide planning, and attempted suicide was nearly 15% in 2017 [2]. Consequently, suicide is the second leading cause of death for AI and AN youths aged 10-24 years, a rate that is 2.5 times higher than the national average [3]. Culturally relevant interventions are critically needed to stem the devastating, reverberating impact that adolescent suicide has on tribal communities and their families [4].

Emerging research has found that some at-risk youth disclose depression symptoms and suicidal ideation on social media channels, including Facebook, Twitter, and Instagram [5]. In a review of college-age social media profiles, 25% displayed references to depression symptoms on their Facebook page [6]. Similarly, in a 1-week period, over 200,000 publicly available tweets included the hashtag “depression,” and approximately 3% of the tweets referenced suicidality [7]. Not only are such references common, they can also be linked to real-life experiences. In one study, participants who displayed references to depression on Facebook also self-reported depression symptoms [8]. Lay media reports and scholarly papers have described tragic cases in which youths’ social media posts have indicated suicidality before attempting or dying by suicide [9]. These public social media disclosures provide new opportunities to identify AI and AN youth (aged 10-24 years) at risk and connect them to appropriate resources and support.

Social media is used frequently by the majority of AI and AN youth (aged 10-24 years) [10]. In a national survey of over 675 AI and AN teens and young adults in 2016, nearly 30% reported seeing concerning posts on a daily basis [10]. Further, multiple previous studies have suggested that teens and young adults from many communities, including AI and AN, are both able to identify social media posts suggesting mental health issues and willing to help intervene [11-14]. Some evidence suggests that AI and AN youth commonly respond when they see a concerning post. According to one survey of AI and AN youth, among those reporting that they saw a concerning post on social media, half indicated they had private messaged the person, 25% commented words of encouragement, one-third talked to the person offline, 20% did *nothing*, 12% *liked* or shared the message, and only 10% sought help from a trusted adult [10]. It is important to note that, although willing, AI and AN youth have indicated a lack of confidence in responding to concerning posts seen on social media [11-14]. There is a need to identify intervention partners to assist youth who observe this kind of content.

Social media connections between youth and adults, such as college students, are increasingly common [15,16], and previous research suggests approaches by which adults may conduct conversations related to sensitive or concerning social media posts. When it comes to discussion of posts seen on their own profiles, teens and young adults in college have reported willingness to discuss these posts, while indicating preferences about who conducts the conversation and how [17]. These preferences include having the conversation with someone the youth knows and that the conversational style be direct and open. Furthermore, teens and young adults in college express willingness to discuss concerning posts seen in electronic media such as email [18]. These findings support the inclusion of known, trusted adults in conversations to assist youth who have seen concerning social media posts. Tribal health educators, who are educators that work in a tribal or urban setting with tribal youth, may be ideally positioned to provide such assistance.

### Objective

Despite the frequency of such concerning posts on social media and the willingness of youth to discuss this content with a known adult, formative research with tribal health educators across the United States found that less than 5% felt adequately prepared to address concerning social media posts [11]. This gap inspired the development of a 1-hour training intervention for adults who work with Native youth: *Responding to Concerning Posts on Social Media*. The aim of this pilot study was to determine the usability, appeal, and impact of the training on adult participants, while identifying components of the training amenable to improvement.

## Methods

### Training Intervention

The three primary learning objectives for the training were to prepare adults who work with Native youth to (1) identify youth who witness concerning social media posts, letting them know they need not respond alone, (2) assess those who see concerning posts and address their concerns, frustration, or fatigue, and (3) confidently implement the *Viewer Care Plan,* a multistep guide for educators to support youth who view or post concerning messages. These objectives were selected after conducting focus groups with 32 AI and AN youths in the Pacific Northwest of the United States, which identified common themes when responding to concerning social media posts: (1) youth frequently responded to concerning posts alone, (2) youth reported complicated barriers to intervening, and (3) youth identified resources and trusted adults they would like to reach out to for additional support [11].

The *Responding to Concerning Posts on Social Media* training intervention included four components: (1) a webinar, (2) a video, (3) handouts, and (4) a coached role-play scenario.

#### Web-Based Training

The Web-based training session was created using Articulate 360 software by Articulate Global, a comprehensive e-learning platform that allows for the creation of responsive and interactive Web-based trainings. The 1-hour Web-based training engaged participants through a self-paced training addressing the three learning objectives. The video included voiced-over narration through slides, which was also available as a readable transcript. The Web-based training session included 9 modules: (1) Welcome, (2) What are concerning posts? (3) Who is this training for? (4) Training goals and overview, (5) 30-minute video training, (6) Training handouts, (7) Community awareness activities, (8) Final review, and (9) A reminder for self-care.

Interactive slides were embedded throughout the Web-based training session to provide opportunities for reflection. The training was self-paced and could be advanced by clicking the *next* or *previous* buttons. A dropdown menu allowed participants to access any part of the training at any point, making access to specific content available for repeated review without needing to start over, and a resource tab gave participants access to downloadable handouts and resources.

Participants were instructed that, under the *supporting materials* tab on the Healthy Native Youth website [19], they could download two activity guides (one for adults and one for youth), which could be used to increase community awareness about concerning posts on social media. The Web-based training session concluded with a reminder for participants to take care of themselves, encouraging them to talk with a trusted friend or family member or a local mental health professional if needed and provided contact information for a clinical psychologist involved with the study.

#### Video

Midway through the Web-based training session, participants viewed a 30-minute video. The video provided an overview of why concerning posts should be taken seriously and shared findings from focus groups with Native youth about their experience seeing and responding to concerning posts on social media. The video included interviews with a Native family, who is from an AI background, and lost a child by suicide. In addition, the video included interviews with mental health professionals, who shared tips and examples for how to support youth and the community.

The video introduced the *Viewer Care Plan* (VCP), a three-step planning and response tool that could be referenced by participants to support youth in their community. The steps of this tool are summarized as follows:

- Step 1 - Start the Conversation: Normalize the topic at community gatherings and school events to raise awareness about concerning posts on social media. Let youth know they can come to you for help, as a trusted adult.
- Step 2 - Listen, Gather Information, and Assess Viewer Experience: If a youth approaches you, praise their efforts to help and assess their well-being. Let them know it can be scary or frustrating to repeatedly see concerning messages. Then, gather information from the viewer. What have they already tried? Who posted the message? How well do you know them? Do you know any trusted adults for the youth who posted the message?
- Step 3 - Plan and Act: Reassure the youth that you will take it from here. If you are comfortable contacting the person who posted the concerning message, use the question, persuade, refer (Question, Persuade, and Refer [QPR]) suicide intervention model. If you would prefer not to, contact another trusted adult to create an appropriate response plan.

The video stressed the importance of adults identifying themselves as trusted adults in the community whom youth can ask for help from.

#### Handouts

The Web-based training session concluded with an overview of the training handouts. These included a two-page summary of concerning posts, examples of concerning messages provided by youth during focus groups, and tips for talking about suicidality; a one-page VCP flyer and tip card; a QPR Institute training flyer to encourage folks to seek additional suicide prevention training; Kognito Web-based role-play simulations to prepare individuals to lead real-life conversations that change lives (free to tribes); a #WeAreConnected suicide prevention rack card; a bullying prevention brochure; and a cyberbullying prevention brochure.

#### Coached Role-Play

After completing the Web-based training session, a subset of participants was invited to participate in a coached role-play scenario. The goal of the role-play was to test and improve participants’ skills as they implemented steps in the VCP. During the role-play, the research team played the part of a youth who had seen a concerning social media post and was reaching out to the participant as a trusted adult for help responding to the post. If the participant forgot or skipped steps during the interaction, the research team offered prompts related to important skills in the VCP (Table 1).

At the end of the interaction, participants were emailed a 3-minute video clip of an exemplar role-play scenario, modeling complete, successful use of the VCP.

**Table 1:** Ideal responses and coaching prompts provided during the role-play scenario.

Viewer care plan skill	Ideal response	Coaching prompt
*Listen carefully and offer reassurance:* Ask youth about the concerning social media post(s). Acknowledge that it can be scary, stressful, and frustrating. If relevant, discuss responder fatigue.	Hi I’m so glad you’re reaching out. Thank you, you are so brave. It can be difficult to know what to do with concerning posts. It’s great that you noticed and reached out to me.	I’m feeling pretty scared and confused. I’m not sure if I should just ignore it, or what. What if they do something, then I’d feel awful. Honestly, this is freaking me out. I want to say something, but just don’t know what.
*Gather information: What have you already tried?* Acknowledge their attempts to provide support.	Have you said or done anything yet in response to the post? Did you like it? Share it? Message them? Have you seen this person posting like this behavior? Is this common for them?	I direct messaged them like 5 times, texted ‘em, and they continue to post depressing stuff I’m over it, I think I’m just going to delete them from my FB. I tried reporting it to Facebook, but it didn’t seem like they did anything-it’s stressing me out. I don’t know who to trust, so I haven’t told anybody.
*Ask about your relationship to the person posting concerning content:* Are they a close friend? Acquaintance? Family member? Avoid interpreting concerning posts to decide whether or not they are meaningful. Move directly to response. *Do you know any adults they would trust to help them?*	Can you tell me a little more about the person who’s posting this content? Is the person posting this a friend, acquaintance, or somebody you know? Do you know any adults or trusted friends who could help this person?	I don’t know if it matters, but I’m like not really good friends with them or anything. I don’t really know them or talk to them in person, but I know their coach from school. Should I tell them?
*Clarify your role:* Be clear about how you can help. Be sure to mention confidentiality and privacy, particularly if you are a mandated reporter. Reassure the viewer that you can take it from here.	You did the right thing by reaching out. I will take it from here. You gave me enough information to follow up with their coach. I will take it from here. We won’t mention your name, they don’t need to know that. So you can feel assured that your privacy will be kept.	Are you going to tell anybody I told you? If ok, I’d like to just keep it between you and I. So, what should I do now? Are you going to reach out to them? I think you’d be way better at knowing what to do than me.
*Follow-up:* Thank the youth who found the concerning messages for reaching out and provide them with resources to feel more confident navigating future concerning posts. The website We R Native has fact sheets and videos for youth on this very topic [20], or Facebook offers safety tools [21]	Are you feeling ok about the situation now that we’ve had the conversation? I want to give you a few resources if you want to learn more about this topic: link to WRN^a^. Facebook also has a tool: link that you can report concerning posts. Again, thank you for reaching out and always know that you can reach out to me. I won’t share your name with anyone else.	If this happens again, are there any places that could help me respond? Isn’t there something on Facebook that I can use to report the post and FB can help them out? I’m not sure if I feel comfortable reaching out if I see this again. Is there something else I can do?

^a^WRN: We R Native.

### Procedures

#### Human Subjects Protections

The study protocol was reviewed and approved by the Seattle Children’s Research Institute Institutional Review Board (IRB) and the Portland Area Indian Health Service IRB. Data collection took place between December 2016 and January 2018.

#### Recruitment

The Northwest Portland Area Indian Health Board recruited participants over a 2-month period using an online recruitment letter that described the training objectives and eligibility criteria. The form was distributed using a Constant Contact listserv and email listserv that reaches over 750 tribal health educators and adolescent health allies across the United States. Those interested in participating were directed to a SurveyMonkey link that confirmed eligibility criteria, followed by an electronic consent form, and a form to collect participant demographics. Demographics collected at this point included: name, tribe, organizational affiliation, state, number of years working with AI and AN youth, types and amount of suicide prevention training, and mailing address for US $15 incentive.

Interest forms were completed by 50 eligible educators living in the Pacific Northwest, including the States of OR, WA, ID, and by 23 educators living in other regions of the United States. Eligible participants included adults (≥18 years old) who mentored and/or regularly interacted with AI and AN youth. All participants indicated they had access to a computer with internet to complete pre-post surveys and watch the video and had access to a mobile phone that could be used to carry out the interactive role-play scenario. In appreciation for their time, participants received US $15 after completing the intervention and US $15 after completing the 6-month follow-up survey.

#### Intervention Delivery

To begin the study, the research team sent participants a welcome email with a link to the Web-based training session and a reminder about using their unique identification. The training was available for participants to complete anytime during the 3-month window. Throughout the duration of the training period, the data manager checked for survey completion on a biweekly basis. Nonresponders were sent tailored reminders to complete the training and pre- and postsurveys up to four times over 3 months.

After completing the Web-based training session with 30-minute training video, participants were randomized using a blocked randomization process, into one of the two study arms (Textbox 1). Those in arm 1 proceeded with completing two follow-up surveys, the first at immediate follow-up and then again at 6 months. Aside from the follow-up surveys, participants randomized to arm 2 received an additional email from the team, scheduling the coached role-play within 2 weeks of the training. Each role-play was conducted with two to four researchers, who viewed the text message exchange in real time using a SMS dashboard and discussed possible responses before sending them to the participant. A short video clip that demonstrates successful application of the VCP during a concerning post scenario was emailed to participants after they completed the role-play.

To collect the 6-month follow-up data, the research team sent participants tailored reminders every 2 weeks, staggered by their training or role-play completion date, over a 3-month period.

**Participants were randomized into two study arms.**

**Study arm 1:**

- Completed 1-hour webinar, including 30-minute video training
- Reviewed training handouts
- Immediate follow-up survey
- Six-month follow-up survey

**Study arm 2:**

- Completed 1-hour webinar, including 30-minute video training
- Reviewed training handouts
- Immediate follow-up survey
- Participated in coached role-play via text message
- Viewed 3-minute video clip of an exemplar role-play scenario
- Six-month follow-up survey

#### Data Collection Instruments and Data Handling

The pre-, post-, and 6-month follow-up survey tools were collaboratively designed by the two research teams. The key outcome measures for the training were changes in concerning post knowledge, intention to intervene, and self-efficacy associated with steps in the VCP. The surveys were collected electronically using Catalyst software. Catalyst is the secure, Web-based survey design and collection software used by the University of Washington and Seattle Children’s Research Institute. The software is approved by the University of Washington IRB and can be configured not to collect respondent IP addresses. Pre- and postsurveys were collected and managed using Catalyst.

The research team randomized eligible participants using three variables (previous suicide prevention training experience, current job role, and self-efficacy), using a cluster randomization by groups, into two study arms. Participants in arm 1 watched the 30-minute training video and reviewed accompanying handouts. Those enrolled in arm 2 watched the 30-minute training video, reviewed the training handouts, and participated in an interactive role-play with a coach that took place after the training, via text message.

### Variables

#### Participants

The presurvey collected participant demographics, including gender, age, state of residence, race and ethnicity, and role in their organization or community. Demographic variables were summarized in terms of frequencies and percentages.

#### Intervention Usability and Appeal

The post survey included three quantitative measures of intervention usability and appeal, including (1) rate the quality of the training video (scale 0-6), (2) rate the quality of the training handouts (scale 0-6), and (3) would you recommend the training to others (yes, no)? At the end of the survey, participants were also asked to provide open-ended feedback on the (1) 1-hour webinar, (2) 30-minute video, (3) the training handouts, (4) a 30-minute coached role-play scenario, (5) the most valuable concepts or skills they acquired during the training, and (6) other ideas or suggestions to improve the training. The team synthesized open-ended responses to identify reoccurring themes.

#### Coached Role-Plays

The postsurvey included two quantitative measures assessing the coached role-play: (1) rate the quality of the coached role-play (scale 0-6), and (2) whether the coached role-play improved the training experience for the participant (yes, no). The investigators also conducted a content analysis of the coached role-play transcripts to evaluate participant adherence to the three steps of the VCP. A total of two trained coders developed a codebook to determine adherence to recommended VCP communication strategies. The research team then viewed the categories and coding system to verify consistency and accuracy, before coding took place. Responses were then analyzed across all role-play participants. The team also synthesized open-ended responses to survey questions, to identify reoccurring themes.

#### Measures of Training Effectiveness

Analysis of measures of effectiveness included paired *t* tests comparing difference of means for self-reported ratings before and after the intervention. The difference of means for self-reported ratings before the intervention and 6 months postintervention was compared using paired *t* tests.

### Analysis

#### Survey Data Analysis

All survey outcome variables were summarized in terms of means and standard deviations. Absolute changes between pre- versus postintervention were calculated and evaluated using a paired *t* test. All reported *P* values were two-sided, and *P*<.05 was used to define statistical significance. Statistical analyses were performed using SAS software, version 9.4 (SAS Institute, Cary, NC) and R software version 2.15.1 [22].

#### Role-Play Content Analysis

A total of two researchers conducted content analysis on the role-play transcripts to assess participants’ adherence to the skills and strategies recommended in the VCP. In addition, the conversational style of each participant was evaluated. To address adherence to the VCP, two trained investigators used a deductive approach in which the VCP informed *a priori* categories, that is, categories were developed before review of data toward capturing items under steps 2 and 3 of the VCP, from listen carefully to resources and tools. Items under step 1 were omitted, as these actions were intended as approaches to make the conversation more likely and would take place before the text message interaction. In addition, *Contact the person at risk* was omitted because the intention was for this step to take place after the initial conversation with the youth who viewed the concerning post.

To evaluate conversational styles, the two researchers used an inductive, open coding technique. An initial review of transcripts led to the development of categories informed by the data. The researchers used an iterative process of reviewing transcripts and refining the codebook until the categories were finalized, resulting in three categories. The first was Directive, in which the participant used an authoritative tone, giving instructions to the youth. The second was Collaborative, in which the participant sought consensus about a plan of action. The third was Noninclusive, in which the participant suggested a plan of action that did not involve the youth. Conversational styles were not mutually exclusive; some participants used more than one. For both content analysis techniques, interrater agreement was calculated using a 20% subsample of transcripts. For Assess Well-being of the Viewer and Gather Information, agreement was 88%. For all other variables, agreement was 100%.

## Results

### Demographics

Eligible prospective participants (N=73) included teachers, coaches, counselors, and tribal health personnel (personnel working with tribal communities), with varying levels of experience responding to someone with suicidal ideation. Most interest form applicants reported having 10 or more years of experience working with Native youth (41/73, 56%), followed by 1 to 5 years (16/73, 22%). Most reported using social media themselves on a daily basis (61/73, 84%) or weekly basis (8/73, 11%). Nearly one-third (22/73, 30%) reported having no formal training in suicide prevention or intervention skills; the remainder identified one or more trainings, including QPR (27/73, 37%), ASIST (27/73, 37%), Kognito (1/73, 1%), SafeTALK (11/73, 15%), Youth Mental Health First Aid (14/73, 19%), or Sources of Strength (2/73, 3%).

The blocked randomization process balanced the two arms in relation to experience working with AI and AN youth, experience using social media, and prior training in suicide prevention. Those who participated in the intervention and completed pre- and postsurveys were primarily female (64/73, 88%) and reported working in youth wellness or prevention; participants also included parents, coaches, teachers, and mental health professionals (Table 2).

**Table 2:** Demographic information for tribal health educator participants.

Demographics			All participants (n=41)		Participants who completed pre- and postsurvey (n=35)		Participants who completed, pre-, post-, and 6-month survey (n=22)
**Gender, n (%)**							
	Male	5 (12)		5 (14)		4 (18)	
	Female	36 (88)		30 (87)		18 (81)	
**Job, n (%)**							
	Teacher or educator	8 (20)		7 (20)		6 (27)	
	Coach	4 (10)		4 (11)		3 (14)	
	Youth wellness or prevention staff	20 (49)		20 (57)		14 (64)	
	Nurse or clinician	2 (5)		2 (6)		3 (14)	
	Mental health professional	6 (15)		6 (17)		5 (23)	
	Parent	7 (17)		6 (17)		4 (18)	
	Other	19 (39)		16 (46)		8 (36)	

### Intervention Usability and Appeal

The immediate postsurvey was completed by 85% (35/41) of participants; the 6-month follow-up was completed by 71% (29/41). Almost half of the eligible participants completed the training intervention associated with their study arm (arm 1: 17/33, 52%; arm 2: 12/35, 34%), and 43% (29/67) completed the 6-month follow-up survey (arm 1: 17/33, 52%; arm 2: 12/35, 34%).

On a 7-point Likert scale (rating 0 as poor and 6 as excellent), participants gave the training video an average score of 5.32 (SD 0.78) and the handouts a score of 5.27 (SD 1.12); all participants indicated they would recommend the training to others.

When asked for open-ended feedback on the training, participants were enthusiastic about its utility and expressed they would recommend it to their peers:

I think the video was VERY well done! The personal experiences in the beginning with the Lukes family really engaged me and brought the severity of the topic home.

I thought it was really good and would like to be able to use it for trainings of others and referral for people. I am a mental health practitioner suicide prevention, intervention, and postvention, and think it would be a valuable tool for me. Good job!

Many offered helpful suggestions to improve the training materials:

The second half of the video could be expanded, to include more focus on Ask About Suicide (ASK) and QPR. Instead they were referred to and mentioned.

Perhaps if a video was made that demonstrated the roleplay (like the one we did on the phone). That was really, really beneficial.

Include additional visuals of emojis and statements used by youth.

Others gave suggestions to support its dissemination in community settings:

Adapt the video for community presentations to educate parents and para-professionals.

Six months after participating in the training, nearly all participants reported benefiting from the training and following its recommendations:

This training is excellent, especially for adults working with youth in contexts beyond social services. Being in Indian County involves many social, economic, educational, and recreational contexts. It is good that it offers training for people to effectively and proactively support our Native youth.

The training was a great tool and discussion starter for folks in our community when I brought up the topic.

### Training Effectiveness

Our pilot evaluation of the effectiveness of the training included positive changes across several measures. A significant positive change was noted across all measures of confidence, including confidence starting a conversation with a youth about concerning posts on social media, intervening when a youth witnesses a concerning post by a family member or acquaintance, and confidence recommending support services to youth who have witnessed or posted a concerning message. Finally, participants reported improved confidence referring an at-risk youth to mental health services. In all cases, the difference of means between pre- and postsurvey was statistically significant, as noted in Table 3.

**Table 3:** Comparison of training effectiveness between pre- versus postintervention.

Variable	Preintervention, mean (SD)	Postintervention, mean (SD)	Change from pre- to postintervention, mean difference (SD)	*P* value
Difficulty supporting youth posting CSMM^a^	1.54 (1.62)	1.31 (1.32)	0.2286 (1.66)	.42
Difficulty supporting youth who saw CSMM	1.31 (1.47)	1.03 (1.27)	0.2857 (1.69)	.32
Confidence starting a conversation with youth about CSMM	4.09 (1.92)	5.17 (0.98)	−1.0857 (2.01)	.003^b^
Confidence intervening effectively when a youth witnesses CSMM by their families or friends	3.63 (1.99)	5.2 (0.96)	−1.5714 (1.99)	<.001^b^
Confidence intervening effectively when a youth witnesses CSMM by their acquaintance	3.29 (1.99)	4.94 (1.19)	−1.6571 (2.01)	<.001^b^
Confidence recommending support services to a youth who witnesses CSMM	4.06 (2.06)	5.43 (0.74)	−1.3714 (2.13)	<.001^b^
Confidence recommending support services to a youth who has posted CSMM	4.37 (1.9)	5.54 (0.66)	−1.1714 (1.99)	.001^b^
Confidence referring a poster of CSMM to a mental health professional within community	4.06 (1.89)	5.34 (0.76)	−1.2857 (1.71)	<.001^b^

^a^CSMM: concerning social media message.

^b^Statistically significant difference.

At the 6-month follow-up, we again identified positive changes in relation to the presurvey. These included sustained positive findings regarding contacting youth who posted concerning messages, confidence starting conversations with youth about concerning social media posts, confidence intervening when a youth witnessed a concerning post, and confidence referring youth who posted or witnessed concerning posts to a mental health provider. Furthermore, there were statistically significant increases in self-reported behavior, including bringing up concerning posts with youth and adults in their community. Each of these changes were significant, as noted in Table 4.

**Table 4:** Comparison of self-reported behavior from pre- to 6 months postintervention.

Variable	Preintervention, mean (SD)	6-month follow-up, mean (SD)	Change from preintervention to 6-month follow-up, mean difference (SD)	*P* value
In the last 6 months, how many times have you brought up CSMM^a^ with youth in your community?	0.89 (0.87)	1.39 (0.86)	−0.5000 (0.86)	.007^b^
In the last 6 months, how many times have you brought up CSMM with adults in your community?	1.04 (0.83)	1.58 (0.99)	−0.5385 (1.10)	.02^b^
What percentage of youth believe it is appropriate to intervene when some posts CSMM?	4.58 (2.89)	5.66 (2.25)	−1.0769 (2.62)	.047^b^
What percentage of adults believe it is appropriate to intervene when some posts CSMM?	5.89 (3.22)	6.77 (2.56)	−0.8846 (2.72)	.11
Confidence contacting a poster of CSMM to assess their risk	3.74 (1.76)	5.12 (0.96)	−1.3846 (1.75)	<.001^b^
In the last 6 months, how many times have you intervened when a youth witnessed CSMM?	1.24 (1.76)	0.74 (0.73)	0.5000 (1.58)	.12
In the last 6 months, how many times have you recommended support services to youth when they witnessed CSMM?	2.58 (3.73)	0.93 (0.98)	1.6538 (3.38)	.02^b^
In the last 6 months, how many times have you referred support services to youth when they posted CSMM?	1.77 (3.15)	0.74 (0.83)	1.0385 (2.66)	.06
Difficulty supporting youth posting CSMM	1.58 (1.63)	1.31 (1.65)	0.2692 (2.15)	.53
Difficulty supporting youth who saw CSMM	1.24 (1.46)	1.00 (1.55)	0.2308 (2.10)	.58
Confidence starting a conversation with youth about CSMM	4.27 (1.72)	5.43 (0.81)	−1.1538 (1.78)	.003^b^
Confidence intervening effectively when a youth witnesses CSMM by their families or friends	3.93 (1.94)	5.43 (0.81)	−1.5000 (2.01)	<.001^b^
Confidence intervening effectively when a youth witnesses CSMM by their acquaintance	3.62 (2.03)	5.00 (1.17)	−1.3846 (2.00)	.001^b^
Confidence recommending support services to a youth who witnesses CSMM	4.31 (1.83)	5.50 (0.77)	−1.1923 (1.79)	.002^b^
Confidence recommending support services to a youth who has posted CSMM	4.54 (1.78)	5.58 (0.71)	−1.0385 (1.68)	.004^b^
Confidence referring a poster of CSMM to a mental health professional within community	4.35 (1.81)	5.54 (0.71)	−1.1923 (1.72)	.002^b^

^a^CSMM: concerning social media message.

^b^Statistically significant difference.

### Coached Role-Plays

Among arm 2 participants, 18 completed the coached role-play, and they gave this training component an average rating of 5.38 (SD 1.02). All reported the role-play improved their training experience. Evaluation of the role-play transcripts found that participants followed some of the VCP guidelines more than others. All participants completed the *Listen Carefully* step. The next most commonly completed step was *Clarify Your Role*, taken by 89% (16/18) of participants, followed by *Ask about Relationship* and *Assess the Well-Being of the Viewer*, both completed by 83% (15/18) of participants. Over half (10/18, 56%) followed the *Gather Information* step. The *Resources and Tools* step was followed least often (8/18, 44%).

A total of three main response styles emerged: Collaborative (11/18, 61%), Directive (8/18, 44%), and Noninclusive (4/18, 22%), with 22% (4/18) of health educators using more than one approach. Collaborative approaches included shared decision-making processes and questions posed to youth confirming their support for a given follow-up plan. For example, one participant asked, “Would you feel comfortable approaching her B-ball coach…about the posts?” Directive approaches, by contrast, involved instructions given to the youth about how to proceed. An example of Directive message was, “Please be sure to contact an adult to support you—and check back in with me.” Finally, when using Noninclusive approaches, participants informed youth of next steps the adult participant would be taking without consensus seeking. One participant said, “I'll take care of following up and I'll talk to the coach or someone else who can help.”

## Discussion

### Principal Findings

This study assessed the usability, appeal, and impact of an hour-long training to prepare adults who work with Native youth to better support teens who post or view concerning messages on social media. Three major findings emerged in addressing this study purpose. First, participants provided feedback that included positive reception of the training. Second, findings supported positive effects of the training on participants’ self-efficacy to address concerning social media posts. Third, analysis of the coached role-plays yielded opportunities for intervention improvements.

Our first major finding was that participants largely regarded the training intervention positively. After completing the training, participants expressed enthusiasm about its content and utility; 100% indicated they would recommend the training to their peers. Participants also offered helpful suggestions to improve the training materials, which have since been used to make updates and enhancements to the webinar, video, and handouts. These findings support the usability and appeal of the intervention.

Our second major finding was that significant improvements were noted across several measures of training efficacy that were retained even at 6 months, including contacting youth who had posted concerning messages, confidence starting conversations with youth, confidence intervening when a youth witnessed a concerning post, and confidence referring youth to a mental health provider. Most importantly, participants reported an increased number of clinical referrals 6 months posttraining, an important marker of training efficacy. These findings are consistent with previous studies using training as an intervention strategy. Previous studies supported Web-based gatekeeper trainings as effective suicide prevention approaches, particularly among adults serving AI and AN communities [4,23-26]. Our findings extend this work by supporting gatekeeper trainings as an effective approach to address concerning social media posts viewed by youth.

Our third main finding was that evaluation of the coached role-play revealed opportunities to improve the training intervention. As in our study, previous research has included a role-play component in gatekeeper trainings to prevent suicide [4,27,28]. Our study is unique in its inclusion of a text message–based role-play, as well as analysis of these conversations’ transcripts to assess training outcomes. For example, analysis of this study’s coached role-play suggested that participants’ adherence to the VCP varied by step. Of note, less than half of participants shared VCP tools and resources. It is possible that participants felt unable to provide adequate resources related to mental health, given that nearly a third did not have previous formal training in suicide prevention or intervention. An alternate possibility is that adults may overlook this step in ensuring that they contact the youth who created the concerning post. Nevertheless, providing resources and tools to the youth who identifies the concerning post may be a critical task. Previous work has suggested that youth viewing concerning posts often do so alone, and they report a need for resources when they encounter such Web-based content [11]. Thus, future research is needed to examine barriers adults may face in offering resources during conversations about concerning posts, such as experience with formal training related to suicide prevention or intervention.

Further, analysis of the coached role-play revealed three communication styles when responding to youth who had viewed concerning posts: Collaborative, Directive, and Noninclusive. One possible explanation for the emergence of these three techniques is professional role and training. For example, a physical education teacher or coach may approach a conversation about a concerning social media post in a collaborative manner to provide support and encourage rapport in absence of specific medical training, whereas a school nurse may be more directive, having training and resources at their fingertips. These findings are important to consider in light of previous research on approaches to conversations about sensitive social media posts. Some evidence suggests that college students have preferences about how to conduct conversations related to concerning social media content [17,18]. Thus, the manner in which a tribal health educator approaches the conversation could have effects on the teen’s well-being, as well as their willingness to collaborate to find help for the youth sharing concerning social media posts. Future research and improvements to the coached role-play component should address youths’ preferred conversational styles and emerging research on effective messages and communication techniques.

Since the completion of the study, the training has been made available to tribal health educators at Healthy Native Youth website [19]. Strong interest and utilization (with over 750 YouTube video views), suggest that this Web-based training is an accessible, convenient format to build self-efficacy for busy adults who work with Native youth.

### Limitations

Several important limitations were present in our study design. This study did not involve a control group and, as such, cannot determine the true effectiveness of the training intervention. The opt-in recruitment strategy also limits the generalizability of our findings, as those who agreed to participate are likely more aware of the importance of this topic than other adults who chose not to participate. In addition, our initial sample size was small; only 85% of participants completed the immediate postsurvey, and just over half of them completed the 6-month follow-up survey. This may have reflected a response bias among our respondents. In a more robust study design, special attention should be given to improve survey response rates and participant retention, especially if conducted over a longer period of follow-up.

### Conclusions

Findings from this pilot study indicate that the *Responding to Concerning Posts on Social Media* training is a promising tool to better prepare adults to intervene and complete the three steps outlined in the VCP: (1) Start the Conversation; (2) Listen, Gather information, and Assess Viewer Experience; and (3) Plan and Act. To our knowledge, this is the first gatekeeper training for adults that provides guidance for responding to concerning posts on social media. Given the frequency of posts by teens and young adults that express suicidality and self-harm, this training may serve as a helpful blueprint for designing similar trainings for other high-risk populations. Additional research with a larger cohort of participants is needed to determine the unique impact of the coached role-play scenario on skill development, and changes in mental health referral rates, behaviors, and skills.

## Abbreviations

AI: American Indian
AN: Alaska Native
IRB: Institutional Review Board
QPR: question, persuade, refer
SAMHSA: Substance Abuse and Mental Health Services Administration
VCP: Viewer Care Plan
